# Frequency-Tagging EEG of Superimposed Social and Non-Social Visual Stimulation Streams Provides No Support for Social Salience Enhancement after Intranasal Oxytocin Administration

**DOI:** 10.3390/brainsci12091224

**Published:** 2022-09-10

**Authors:** Zhiling Qiao, Stephanie Van der Donck, Matthijs Moerkerke, Tereza Dlhosova, Sofie Vettori, Milena Dzhelyova, Ruud van Winkel, Kaat Alaerts, Bart Boets

**Affiliations:** 1 Center for Clinical Psychiatry, Department of Neuroscience, KU Leuven, 3000 Leuven, Belgium; 2 Center for Developmental Psychiatry, Department of Neurosciences, KU Leuven, 3000 Leuven, Belgium; 3 Leuven Autism Research (LAuRes), KU Leuven, 3000 Leuven, Belgium; 4 Department of Psychology, Faculty of Arts, Masaryk University, 60200 Brno, Czech Republic; 5 Institute of Cognitive Sciences Marc Jeannerod, UMR5229, CNRS, University Claude Bernard Lyon1, 69675 Bron, France; 6 Institute of Research in Psychological Sciences, Université de Louvain, 1348 Louvain-la-Neuve, Belgium; 7 UPC, KU Leuven, 3000 Leuven, Belgium; 8 Department of Psychiatry and Psychology, School for Mental Health and Neuroscience, Maastricht University, 6211 Maastricht, The Netherlands; 9 Research Group for Neurorehabilitation, Department of Rehabilitation Sciences, KU Leuven, 3000 Leuven, Belgium

**Keywords:** EEG, social stimuli processing, frequency-tagging EEG, oxytocin

## Abstract

The social salience hypothesis proposes that the neuropeptide oxytocin (OT) can impact human social behavior by modulating the salience of social cues. Here, frequency-tagging EEG was used to quantify the neural responses to social versus non-social stimuli while administering a single dose of OT (24 IU) versus placebo treatment. Specifically, two streams of faces and houses were superimposed on one another, with each stream of stimuli tagged with a particular presentation rate (i.e., 6 and 7.5 Hz or vice versa). These distinctive frequency tags allowed unambiguously disentangling and objectively quantifying the respective neural responses elicited by the different streams of stimuli. This study involved a double-blind, placebo-controlled, cross-over trial with 31 healthy adult men. Based on four trials of 60 s, we detected robust frequency-tagged neural responses in each individual, with entrainment to faces being more pronounced in lateral occipito-temporal regions and entrainment to houses being focused in medial occipital regions. However, contrary to our expectation, a single dose of OT did not modulate these stimulus-driven neural responses, not in terms of enhanced social processing nor in terms of generally enhanced information salience. Bayesian analyses formally confirmed these null findings. Possibly, the baseline ceiling level performance of these neurotypical adult participants as well as the personal irrelevance of the applied stimulation streams might have hindered the observation of any OT effect.

## 1. Introduction

### 1.1. Oxytocin as a Complex Modulator of Complex Social Behavior

The modulatory effect of the neuropeptide oxytocin (OT) has been implicated in a wide range of social behaviors in humans. For example, intranasal OT administration has been shown to enhance prosocial behavior, including cooperative trust, generosity and affiliation [1,2,3,4,5], and may play an important role in social cognition, as it has been found to improve emotion recognition [6,7], mentalizing [8] and eye contact [9,10]. These findings of a positive effect of OT on socio-cognitive abilities and social functioning stimulated a growing number of studies evaluating its therapeutic applications in a variety of neuropsychiatric disorders, such as autism spectrum disorder [11,12,13,14], schizophrenia [15] and personality disorder [16]. However, the picture is complicated by studies revealing that the effects of OT are not uniformly positive or prosocial. For instance, Shamay-Tsoory et al. [17] demonstrated that OT might increase feelings of gloating and envy, and De Dreu et al. [3] found that OT enhanced defensive aggression toward competing out-group members. Elevated plasma OT has also been associated with interpersonal difficulties and relational distress [18,19].

### 1.2. The Social Salience Hypothesis of OT

The social salience hypothesis aims at reconciling these conflicting observations and suggests that OT increases the salience of social cues regardless of valence [20] but is dependent on context and individual characteristics, such as gender, personality, attachment style and psychopathology [21]. Indeed, with regard to context dependency, OT has been shown to enhance prosocial behavior (e.g., trust and cooperation) when interacting with intra-group members, but not when interacting with out-group members [3] or when competing with others [17]. The context-dependency of OT has also been evidenced by showing that it selectively impacts the processing of social but not non-social stimuli: it enhances recognition memory for faces but not for non-social stimuli [22]; it improves the detection of socially-related words but not of other words [23]; it facilitates learning from social feedback but not from non-social feedback [24]; it affects arousal ratings of images depicting humans but not of pictures depicting animals [25].

While the social salience hypothesis can explain many of these contrasting findings, the mechanism of the selective and modulatory effect of OT on social processing is only poorly understood. Shamay-Tsoory and Abu-Akel [21] proposed that OT may increase the salience of social cues by altering attentional neural mechanisms. Indeed, at a behavioral level, OT has been found to increase attention orientation toward faces, in particular, expressive faces [26,27], and to increase looking times toward the eye region [10,28]. Enhanced stimulus-induced pupil dilation in response to social stimuli has also been demonstrated after OT administration, indicating enhanced overt attention toward social stimuli [29]. A recent study investigated the modulatory effects of OT on the processing of social versus non-social stimuli via binocular rivalry. The authors showed a robust effect of intranasal OT on increasing the salience of faces but also a modest (non-significant) increase in the salience of non-social stimuli [30]. At the neural level, OT has been shown to selectively enhance fMRI brain activities in response to faces versus non-social stimuli in early visual areas [31]. In addition, reduced activation of the amygdala has been evidenced in response to both socially and non-socially threatening images, but with a more prominent effect on social stimuli [32]. Event-related potential (ERP) studies reported increased amplitudes of the N170 [33], the vertex positive potential (VPP) and late positive potential (LPP) [34] in response to expressive faces after OT versus placebo administration, demonstrating enhancement of social attention at both the early and late stages of attentional processing. Yet, investigating the effect of OT on the salience of different facial features (eyes, nose, mouth) of neutral faces revealed no OT-associated effects, as indicated by the latencies and amplitudes of ERP components P100, N170 and the earlier posterior negativity (EPN) [35]. Thus, taken together, both behavioral and neural findings generally suggest that OT modulates overt as well as covert attentional processing of social information, in particular faces.

### 1.3. Pinpointing the Neural Salience of Social Versus Non-Social Information via Frequency-Tagging EEG

We combined fast periodic visual stimulation with frequency-tagging electroencephalography (EEG) to investigate the modulatory effect of a single dose of OT on the attentional processing of simultaneously presented social and non-social stimuli at the neural level. Frequency-tagging EEG is based on the concept that the brain synchronizes its activities to periodically flickering stimuli [36,37]. This technique is particularly suitable for studying visual attention as it provides an objective and reliable measure of neural activity with high signal-to-noise (SNR), which is unambiguously related to the specific stimulus, even when multiple stimuli are presented at the same time [38]. In a previous study on social attention in 8–12-year-old boys with autism spectrum disorder (ASD), Vettori et al. [38] combined frequency-tagging EEG with eye-tracking to study both covert and overt social attention, respectively. The authors presented children with streams of faces (social stimuli) and houses (non-social stimuli), each tagged at a particular presentation rate. By simultaneously presenting these stimuli streams next to each other, they found a significantly reduced social bias in children with ASD, reflected in reduced looking times to the faces, but, most importantly, by reduced neural responses toward the stream of faces [38]. As the possible influence of preferential looking, disengagement and spatial attention could not be ruled out, in a follow-up study [39], the authors presented the same streams of stimuli (i.e., faces and houses) spatially superimposed to control for these potential confounds. Prior research using frequency-tagging EEG in combination with superimposed stimuli has indeed evidenced that attention can modulate neural processing in a nonspatial way: even when, for instance, objects are spatially overlapping, selectively attending to one of the stimuli streams has been demonstrated to elicit enhanced neural responses [40,41,42,43,44,45]. In the study of Vettori et al. [39], neurotypical boys showed a socially driven modulation of the neural response, with stronger frequency-tagged brain responses to faces than those to houses, particularly in lateral occipito-temporal regions, whereas responses to houses were stronger in medial occipital regions. Yet, in the ASD group, this modulation of the response was not observed in any of the regions.

Given the high test–retest reliability of frequency-tagging paradigms and their ability to sensitively pinpoint differences in the processing of various socio-communicative cues in infants [46], children [38,47,48,49] and adults [50,51], this method is thought to be quite suitable for monitoring subtle changes in the neural responses to social and non-social cues, as induced by intranasal OT administration [52].

### 1.4. The Present Study

In this study, we recorded EEG signals while participants were simultaneously presented with two streams of images of faces (i.e., social category) and houses (i.e., non-social category) superimposed on one another on the screen [39] to investigate the effect of a single dose of OT on the salience of social and non-social cues. Since the two types of stimuli were tagged at different frequency rates, responses to both stimulus streams could separately be analyzed even though they were displayed simultaneously and superimposed. Moreover, in line with Vettori et al. [39] and with established neuroanatomical models on visual categorization, we expect that faces will preferentially engage lateral occipito-temporal areas (in particular, the fusiform gyrus) [53,54,55,56], whereas houses may rather mobilize medial regions of the ventral occipitotemporal cortex, such as the parahippocampal gyrus and collateral sulcus [45,57,58,59,60]. Thirty-one healthy male adults participated in a double-blind, within-subjects, placebo (PL)-controlled, cross-over clinical trial, where they were randomly assigned to receive either a single dose of OT or placebo during two test sessions separated by a two-week interval. Following the social salience hypothesis [21,30], after exogenous OT administration, we would expect selectively increased frequency-tagged EEG responses to faces but not to houses.

## 2. Materials and Methods

### 2.1. Participants

As this study was conducted as part of a larger study on the effects of OT, participants were identical to those recruited in Van der Donck et al. [52]: 31 18–32-year-old (mean age = 22.81 ± 2.38 years) healthy male participants, all right-handed and with normal or corrected to normal vision. Inclusion criteria were the absence of any diagnosed genetic, psychiatric or neurological disorders in the participant or a first-degree relative. To avoid possible differences in terms of gender in response to OT administration [61,62], only male participants were included.

This study was approved by the Medical Ethical Committee of the university hospital. Written informed consent was obtained from each participant prior to the study, and each participant received monetary compensation for their participation.

### 2.2. Study Design

Identical to Van der Donck et al. [52], the study consisted of a randomized, double-blind, within-subjects, PL-controlled, cross-over clinical trial. Participants took part in two identical sessions—apart from the nasal spray they received—at exactly the same time of the day, two weeks apart. Participants were randomly assigned to either receive the PL spray (saline solution of sodium chloride in water) in the first session and the OT spray (Syntocinon^®^, Sigma Tau, Pomezia, Italy) in the second session or vice versa.

A single dose of 24 international units (IU) of OT was applied and administered via three puffs of 4 IU per nostril [63,64]. Based on previous studies [63,65,66], we incorporated a time interval of 30 min between the intranasal administration of OT and the start of the EEG paradigm in order to test during peak OT concentrations. Potential side effects due to the administration of OT were monitored throughout the entire session and reported in Van der Donck et al. [52].

### 2.3. Procedure

Participants were seated in front of an LCD 24-in computer screen with an 80 cm viewing distance in a dimly lit room and were instructed to maintain a constant distance during EEG recording. A screen with a 60 Hz refresh rate was used to ensure that the refresh rate was an integer multiple of the presentation frequencies. Using a custom-built Java script, stimuli were presented on the screen through sinusoidal contrast modulation on a light grey background. In total, four sequences—each with a duration of 60 s—were administered, resulting in a total stimulus presentation of 4 min. The order of the four sequences was randomized for each participant. Runs were started when participants were attentively looking at the screen. In between runs, participants could take as much rest as needed.

#### 2.3.1. Stimuli

**Figure 1:**
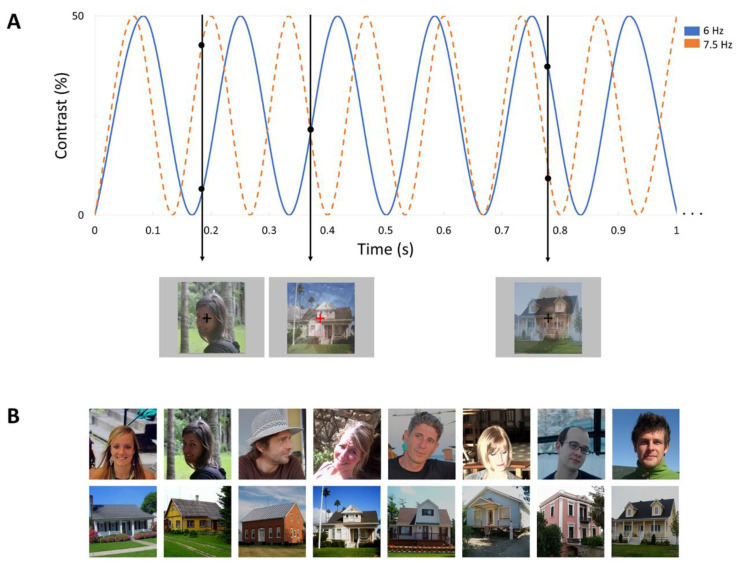
Experimental design. (**A**) Illustration of a stimulation sequence, with houses presented at 6 Hz and faces presented at 7.5 Hz. The presentation rates (6 and 7.5 Hz) were counterbalanced across the two stimulus types. In total, four sequences of 60 s were administered. Contrast of all images was modulated from 0 to 50%. The first black arrow indicates what was presented at 0.18 s: the second face was presented at around 40% contrast at this timepoint, whereas the second house was presented at around 10% contrast. (**B**) Examples of selected faces and houses.

The stimuli sets consisted of 48 widely varied images of houses and 48 images of faces, selected from [60] and [67]. Spectral analyses indicated that images of houses have more contrast in higher spatial frequencies and cardinal orientations (for details on the amplitude spectra of faces and houses, see [39]). Faces and houses were superimposed on one another, with a fixation cross presented in the center of the images (Figure 1). All stimuli were set to a size of 250 × 250 pixels and had equalized mean pixel luminance and contrast during the presentation. The stimuli subtended around 2.61 × 2.77° of visual angle, shown at a viewing distance of 80 cm from the screen and at a resolution of 1920 × 1200.

#### 2.3.2. Frequency Tagging Paradigm

The design was similar to the study of Vettori et al. [39]. Each sequence consisted of two streams of simultaneously presented images of faces and houses, which were superimposed at the same position and shown at the center of the screen. The contrast of the images periodically increased and decreased between 0 and 50% (see Figure 1). Throughout a sequence, images of one stimulus category were presented at 6 Hz and images of the other category at 7.5 Hz, or vice versa, which was counterbalanced through the whole task. All images were drawn randomly from their respective categories, cycling through all available images before any image repetition. In order to guarantee a constant level of attention, an orthogonal task was implemented. Participants were instructed to focus on a fixation cross presented in the center of the images and to press a key whenever they detected brief (300 ms) color changes (black to red) of this fixation cross. The color changes occurred randomly 15 times within every sequence.

#### 2.3.3. EEG Acquisition

EEG was recorded using a BioSemi Active Two amplifier system with 64 Ag/AgCl electrodes. Two additional electrodes (CMS, common mode sense, and DRL, driven right leg) were used as reference and ground electrodes. We placed two external electrodes at the outer canthi of the eyes to record horizontal eye movements and one external electrode above and one below the right eye to record vertical eye movements. A continuous EEG signal was sampled at 512 Hz.

### 2.4. Data Analysis

#### 2.4.1. EEG Analysis

- Preprocessing

All data processing was performed using Letswave 6 (https://www.letswave.org/, accessed on 29 July 2021) and MATLAB 2021 (MathWorks). The EEG signal was cropped in 68-s segments (2 s before and 6 s after each sequence), bandpass filtered (0.1 to 100 Hz) using a fourth-order Butterworth filter, and downsampled to 256 Hz. For three participants who blinked on average more than 1.5 *SD* above the mean (the average number of blinks per second across participants = 0.09, SD = 0.08), we applied independent component analysis via the runica algorithm [68] and removed the component that accounted for most of the variance. Noisy channels were linearly interpolated using the three spatially nearest electrodes (not more than 5% of the electrodes, i.e., three electrodes, were interpolated). All data segments were re-referenced to a common average reference.

- Frequency-Domain Analysis

The preprocessed segments were further cropped to contain an integer number of 1.5 Hz cycles (i.e., the largest common divisor of both 6 and 7.5 Hz), beginning at the start of the sequence and until 59.3867 s (15,203 time bins). In order to reduce EEG activity out-of-phase with the stimulation (i.e., noise), the resulting segments were averaged per presentation rate for each stimulus type under each treatment condition. Thereafter, a Fast Fourier transform (FFT) was applied to transform the data from the time domain into the frequency domain, and the amplitude spectrum was computed with a spectral resolution of 0.017 Hz (1/59.3867 s), resulting in a very high SNR [37,69].

Since the brain synchronizes its activity to the presentation rates of the stimuli, the recorded EEG contained a signal at frequencies that were integer multiples (harmonics) of the presentation rates (6 Hz and 7.5 Hz). Two measures were used to describe the responses: SNR to visualize the data [70] and baseline-corrected amplitudes to quantify the data [67]. We computed SNR spectra by dividing the amplitude value of the target frequency bin by the average amplitude of the 20 neighboring frequency bins (i.e., 10 bins on each side of the target frequency bin, excluding the immediately neighboring bins and the two bins with the most extreme values). Baseline-corrected amplitudes were computed by subtracting the amplitude of the frequency bin of interest by the average amplitude level of the 20 surrounding bins.

To define the harmonics that were significantly above the noise level, the FFT data -averaged across all participants and across all electrodes in the regions of interest (ROIs)- were transformed into Z-scores by dividing the difference between the amplitude at each frequency bin and the mean amplitude of the corresponding 20 surrounding bins, by the SD of the amplitudes in these 20 surrounding bins [70,71,72]. Harmonics were considered significant and relevant until the Z-score for two consecutive harmonics no longer exceeded 1.64 (*p* < 0.05). Consequently, the brain responses to faces and houses were quantified by summing the baseline-subtracted responses for the first two harmonics: 6 and 12 Hz, and 7.5 and 15 Hz, for the 6 Hz and 7.5 Hz stimulation frequencies, respectively. This allowed us to obtain the neural responses to each stimulus type at each presentation rate per treatment condition.

Additionally, we conducted the analyses at the individual level by first cropping the raw FFT spectrum into segments centered at the target frequencies and their harmonics, surrounded by 22 neighboring bins on each side that represented the noise level. These spectra were then summed across the significant harmonics of each target frequency and transformed into Z-score.

- Defining ROIs

Following previous studies [38,39] and visually confirmed by inspecting the topographical maps in both treatment conditions, we identified three ROIs: left occipito-temporal (LOT: P7, P9, PO7), medial occipital (MO: Iz, Oz, O1, O2) and right occipito-temporal (ROT: P8, P10, PO8) regions.

#### 2.4.2. Orthogonal Data Analysis

Both accuracy and reaction times were calculated to evaluate the detection of the fixation cross-color changes. A key press was considered correct if it occurred in the 100 to 2000 ms time window following an actual color change. Hence, for these correct responses, the corresponding reaction time was calculated. Accuracies and reaction times were averaged across the four sequences, resulting in an overall accuracy and reaction time per participant per treatment condition. One participant was excluded from this analysis due to technical difficulties resulting in the loss of the numeric triggers for the fixation cross color change and key press recording.

#### 2.4.3. Statistical Analysis

Linear mixed models (LMM; “afex” package version 1.1.0) [73] were performed in R version 4.1.3 [74] with treatment condition (OT, PL), stimulus type (face, house) and ROI (LOT, ROT, MO) as fixed effects. As half of the participants started with the PL condition and the other half received OT during their first session, the session order was included as a nuisance covariate to adjust for its potential effect on neural responses. In order to account for the clustered nature of the repeated measurements, the random intercept and slope were included for each of the fixed factors per participant. A total of 26 of 744 data points were detected as outliers using the median absolute deviation and removed. Post-hoc Z-tests and holm-corrected *p*-values were used to compare means using the emmeans package version 1.7.3 [75].

LMMs were also performed on the accuracy and reaction times of the orthogonal task. Treatment condition (OT, PL) was included as the fixed factor and session order was included as a nuisance covariate.

When no significant treatment effect was revealed by the LMMs, we computed Bayes Factors (BFs) to validate the null findings (“BayesFactor” package version 0.9.12–4.3 [76]). To compute the BFs, we compared the fitting of the model as described in the LMMs versus the fitting of the same model without the factor “treatment condition”. Consistent with the classification scheme [77,78], a BF-value of 1 indicates that the data are equally likely under either model, whereas values between 3 and 10 are taken as moderate evidence for the model in the numerator, values between 10 and 30 as strong evidence, and values between 30 and 100 as very strong evidence. The inverse of these cut-offs provides evidence for the model in the denominator. Additionally, mean posterior differences between the OT and PL treatment conditions and their 95% highest density interval (HDI) were calculated for each stimulus type in each ROI, as well as for the accuracy and reaction time of the orthogonal task.

## 3. Results

### 3.1. Orthogonal Task Performance

LMM analyses on the orthogonal task revealed equal performances during the PL and OT conditions, both in terms of accuracy (*F*(1, 29) = 0.21, *p* = 0.65, mean accuracy = 94% ± 2% under PL treatment condition, mean accuracy = 95% ± 3% under OT treatment condition) and in terms of reaction time (*F*(1, 29) = 0.01, *p* = 0.90, mean reaction time = 0.42 ± 0.01 sec under both PL and OT treatment conditions). These results indicated a similar level of attention under the two treatment conditions.

BF analyses further provided moderate evidence for the absence of a treatment effect on the accuracy (BF_acc_ = 0.279) and reaction time (BF_rt_ = 0.265). Results of mean posterior differences confirmed that participants scored virtually identical in detecting the color changes of the fixation cross, both in terms of accuracy (mean posterior difference = 0.008; 95% HDI = −0.027 0.044]) and in terms of reaction time (mean posterior difference = 0.001; 95% HDI = [−0.013 0.014]), under the OT versus the PL conditions.

### 3.2. Neural Responses

#### 3.2.1. Test–Retest Reliability and Power Analysis

A Pearson correlation demonstrated a high test–retest reliability of the brain responses obtained in our study (with between-session correlations of *r* = 0.91 for faces and *r* = 0.89 for houses; all *p* < 0.001). According to the average correlation (*r* = 0.90), we conducted a power analysis with G*Power 3 [79], revealing a power of 0.95 to detect either a main effect of treatment or treatment by stimulus type interaction, even for a small effect size (d = 0.3). This indicated that the current study design was of adequate power to detect true differences.

#### 3.2.2. Oxytocin Modulation Effect on Social and Non-Social Stimuli

**Figure 2:**
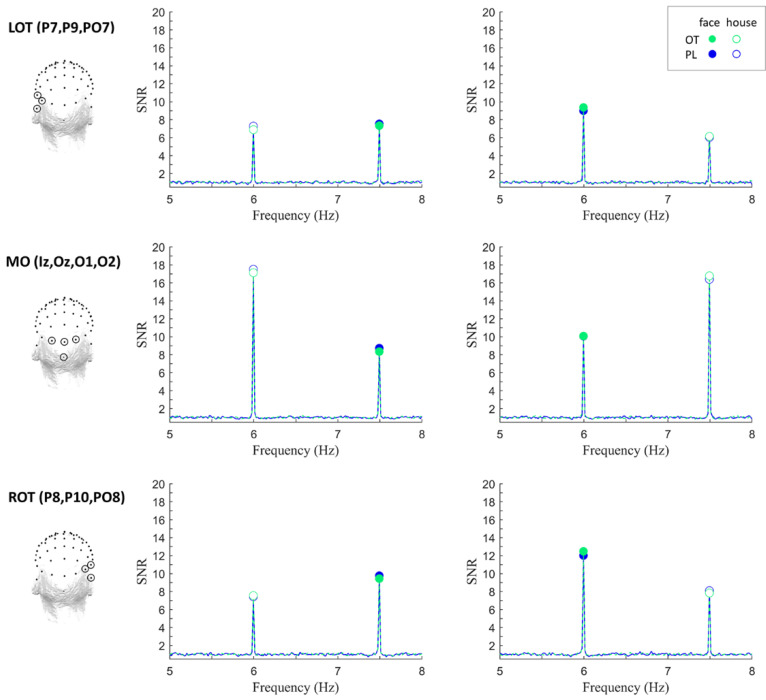
Grand-averaged signal-to-noise ratio (SNR) spectra at the first harmonic of the target frequencies. Data were plotted for the left occipito-temporal (LOT: P7, P9, PO7) region (upper panel), the medial occipital (MO: Iz, Oz, O1, O2) region (middle panel) and the right occipito-temporal (ROT: P8, P10, PO8) region (lower panel). Full circles illustrate the neural responses to faces (i.e., on the left faces were presented at 7.5 Hz, while the right faces were presented at 6 Hz), and empty circles illustrate the neural responses to houses (i.e., on the left houses were presented at 6 Hz, while on the right houses were presented at 7.5 Hz). Green lines indicate the oxytocin treatment, while blue lines indicate the placebo treatment.

**Figure 3:**
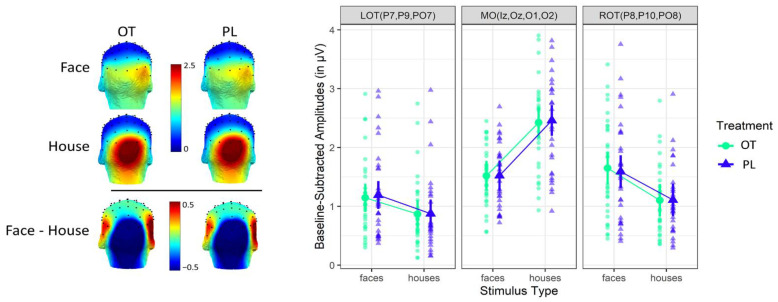
Left: scalp distribution of the EEG signal based on baseline-subtracted amplitudes in µV. Right: grand-averaged baseline-subtracted amplitudes at each ROI for each stimulus type under each treatment condition. Statistical analysis showed a significant interaction between stimulus type and ROI.

We observed robust frequency-tagged neural responses to faces and houses in the three ROIs under each treatment condition. Figure 2 displays the SNR spectra at the first harmonic of target frequencies, and Figure 3 displays scalp distributions and averaged baseline-subtracted amplitudes. Analysis at the individual subject level revealed that all participants showed significant neural responses (i.e., Z-scores > 1.64, *p* < 0.05) to faces and houses in the three ROIs at each presentation rate per treatment condition.

Results of LMM analyses on neural responses are listed in Table 1. There was no significant main effect of treatment, nor a significant interaction effect between treatment and stimulus type, treatment and ROI, and treatment, stimulus type and ROI. Results revealed a significant main effect of ROI, with larger responses in the MO region than in the LOT and ROT regions and larger responses in the ROT region than in the LOT region. In line with the neurotypical data of Vettori et al. [39], these effects were qualified by a highly significant interaction effect between stimulus type and ROI. Post-hoc testing revealed that for faces, the responses were larger in the ROT and MO regions as compared to the LOT region, while for houses, the responses were larger in the MO region than in both occipito-temporal regions. In the left and right OT regions, the responses were larger for faces than for houses, while in the MO region, the responses were larger for houses than for faces.

Visual inspection of Figure 3 provided the decisive evidence for an absence of treatment effect, which was also convincingly confirmed by the BF analysis: BF < 0.001. Participants score virtually identical in response to faces in LOT (−0.018 μV; 95% HDI = [−0.132 0.093]), MO (−0.016 μV; 95% HDI = [−0.132 0.099]) and ROT regions (0.034 μV; 95% HDI = [−0.080 0.149]), and to houses in LOT (0.018 μV; 95% HDI = [−0.093 0.132]), MO (0.016 μV; 95% HDI = [−0.099 0.132]) and ROT regions (−0.034 μV; 95% HDI = [−0.149 0.080]), under the OT versus the PL conditions.

## 4. Discussion

### 4.1. Quantifying Implicit Attentional Processing via Frequency-Tagging EEG

According to the social salience framework [21], exogenous OT administration would selectively increase the salience of social cues by altering attentional neural mechanisms, irrespective of the valence of the stimuli. Here, we quantified implicit attentional neural responses toward social versus non-social stimuli by frequency-tagging superimposed streams of faces and houses while recording EEG. As brain activity synchronizes at exactly the same frequency of the stimulation, this approach allows monitoring neural responses, even to multiple simultaneously presented stimuli. Importantly, these neural responses were implicit, as no active behavior of the participants was required, except for looking at the screen. Neural responses were quantified in an objective way as they were locked to the specific presentation rates of the stimuli [37,69]. Furthermore, by presenting superimposed simultaneous stimulation streams, we ruled out any possible confounds in terms of looking patterns, attentional disengagement and spatial attention. In general, our findings confirm the original findings of Vettori et al. [39], showing that frequency-tagged neural responses in neurotypical participants are modulated by the social versus the non-social character of the stimuli, with faces evoking stronger brain responses than houses over lateral occipito-temporal regions and houses evoking larger brain responses than faces over medial occipital regions. However, neural responses were identical under both treatment conditions (OT and PL). Thus, we did not observe any OT-induced enhancement of attentional allocation, not selectively toward social stimuli, nor generally toward both social and non-social stimulus categories, thereby arguing against the enhanced social salience account of OT.

### 4.2. Topographical Selectivity for Responses toward Houses Versus Faces

We observed significantly higher responses to faces versus houses over lateral occipito-temporal channels (i.e., LOT and ROT regions) and stronger responses to houses versus faces over medial occipital channels (i.e., MO region). These results are consistent with previous observations suggesting a spatial dissociation between face- and house-selective responses [45,80]. Indeed, while houses are associated with responses in medial occipito-temporal brain regions, such as the medial temporal gyrus, parahippocampal gyrus and collateral sulcus, faces are associated with responses in lateral ventral occipito-temporal brain regions, such as the lateral fusiform gyrus and inferior occipital gyrus [57,58,59,81]. Combining frequency-tagged MEG with ROIs defined based on individual fMRI localizers, Baldauf and Desimone [45] evidenced these category-selective responses to superimposed images of faces and houses: while selective attention allocation to faces increased the responses in the fusiform gyrus, selective attention allocation to houses enlarged the responses in the parahippocampal gyrus. Moreover, these different category-selective responses to faces and houses have been demonstrated in scalp EEG [82], even when the presented stimuli are overlapping [39].

### 4.3. Strong Biomarker Characteristics but No Treatment Effect

One may wonder whether our paradigm would lack the sensitivity to detect subtle changes throughout an intervention. By quantifying the neural responses at the individual level, we found that all participants yielded robust individual responses (i.e., Z-scores >1.64, *p* < 0.05) to each stimulus type, irrespective of the treatment condition, indicating the sensitivity of the paradigm. High test–retest reliability was also demonstrated for the neural responses obtained in our study (i.e., between-session correlations for faces and houses were 0.91 and 0.89, respectively), consistent with what has been evidenced in previous studies using frequency-tagging EEG paradigms [52,83]. Furthermore, the very same paradigm has been shown to differentiate successfully between boys with and without ASD [39], a neurodevelopmental disorder characterized by impairments in social communication and interaction, often including a reduced orientation toward social stimuli. Thus, against this background, the evidenced reliability and sensitivity at the individual-subject level in our study would have made the frequency-tagging EEG paradigm a perfectly suited biomarker to quantify the neural responses to social versus non-social information and to monitor a potential modulatory effect of OT treatment [84].

However, we did not detect any OT-associated modulation effect on attention. If OT would uniquely enhance the salience of social stimuli, this would have been reflected in a selective increase in response to faces with OT treatment as compared to placebo. However, this is not the case in our study, thereby arguing against the social salience account. Alternatively, if OT would increase attention allocation in general, this may have been reflected in increased response amplitudes for both the stream of faces and houses, thus regardless of the social nature of the stimulation. However, we did not observe a significant main effect of treatment, thereby arguing against a more general OT modulation effect on attention. Non-significant *p* values may be hard to interpret, as they may either indicate a lack of sensitivity to detect existing differences (e.g., the sample size was too small) or reflect actual null results [85]. However, our study comprised 31 participants, and the power analysis indicated excellent power, especially because of the exceptionally high test-retest reliability of our measurements. Moreover, an additional Bayesian analysis [85,86] convincingly confirmed the null hypothesis and the absence of an OT effect on attentional processing, neither for social nor for non-social stimuli.

### 4.4. Lack of Treatment Effects Due to Baseline Ceiling Levels?

In our parallel study with an overlapping subject sample, Van der Donck et al. [52] quantified OT effects on the implicit neural sensitivity for positive (i.e., happy) and negative (i.e., angry and fearful) facial expressions. Here, results showed no OT enhancement of emotional salience, neither in the frequency nor in the time domain. This also argues against the social salience hypothesis because, according to this framework, generally enhanced neural responses to the three displayed facial expressions should have been detected. Possibly, the absence of any modulatory OT effect in our particular sample could be explained by the fact that OT may have a more limited role in augmenting social salience in individuals with high baseline capabilities, as may be the case in this sample of neurotypical adults. Indeed, selective improvements of, for instance, empathic accuracy or mentalizing ability have been found in the less socially capable individuals [87] or in those with lower empathy scores at baseline [88], indicating that OT’s modulatory effect of salience may vary as a function of baseline individual differences such as gender, personality traits and degree of psychopathology [21].

### 4.5. Lack of Treatment Effects Due to Lack of Personal Relevance?

The General Approach–Avoidance Hypothesis of OT (GAAO) [89] posits that OT acts on the mesocorticolimbic circuitry of approach motivation as well as the cortico-amygdala circuitry of withdrawal/avoidance motivation. As the neural substrates of “social” versus “non-social” approach and avoidance are not distinct from each other, OT is postulated to exert a modulatory effect both on social and non-social behaviors. Previous studies have shown that OT may indeed enhance the attentional salience of many cues in the environment, not specifically because they are social but rather because they are emotionally evocative and personally relevant [89]. Indeed, for individuals high in anxiety sensitivity for whom negative emotion (anxious arousal) was motivationally-relevant, OT specifically reduced behavioral avoidance of negatively-valenced stimuli, irrespective of whether these stimuli were social or non-social [90]. Likewise, OT has been shown to specifically amplify approach-related motivational salience of stimuli that were self-reported to have high personal relevance without regard to the stimuli’s social context or affective valence (positive/negative) [91]. Against this background, the absence of OT effects in our study can be understood as a consequence of using social (i.e., faces) and non-social (i.e., houses) stimuli that were not personally relevant and emotionally evocative, and therefore, OT was not likely to shape the neural responses toward these stimuli.

### 4.6. Lack of Treatment Effect Because of Lack of OT Impact?

Alternatively, intranasal OT may have a more limited direct effect on social processing than previously assumed [86,92,93]. For instance, Tabak et al. [86] investigated the effect of a single dose of OT on a series of social outcomes, including empathic concern, social versus non-social working memory, deception detection, the influence of interpersonal distance on perceptions of trustworthiness and threat ratings, bystander helping, and supportive interaction and conflict, in undergraduate students. The results revealed no main effects of OT. These null findings were further validated by equivalence testing and Bayesian hypothesis testing, indicating that 47–83% of their results had enough sensitivity to confirm the absence of a main effect statistically. Lane et al. [94] retrieved studies buried in their drawer and presented a complete overview of the OT research performed in their laboratory. The authors found that only one out of 25 tasks showed a statistically significant main effect of intranasal OT, and only 5 out of 25 tasks showed a significant interaction effect, including the treatment condition (OT vs. PL). In contrast, a Bayesian meta-analysis on the efficacy of intranasal OT to improve areas of social cognition and non-social neurocognition in schizophrenia indicated that OT might have selective effects on high-level social cognition [95]. Thus, even though a large number of studies provide compelling evidence for OT’s modulatory effect on a vast array of complex social cognition behavior in humans, both in neurotypical and in patient populations [3,5,8,12,96,97,98], accumulating null results also start raising concerns about the reproducibility and validity of these findings. No matter whether the inconsistent evidence may be attributed to the fact that OT’s effects are too small to detect or too selective, for instance, in terms of social cognitions [95] or individual characteristics [21], a more comprehensive and complementary consideration of methodology, theory and reproducibility will be necessary [85,94,99]. The use of reliable, objective and well-specified measurement instruments as in this study will hopefully contribute to this objective.

## 5. Conclusions

To conclude, combining frequency-tagging EEG with superimposed streams of widely variable images of faces and houses, we investigated the effects of a single dose of intranasal OT on the attentional neural responses toward social versus non-social stimuli. In line with previous research, stronger responses to faces were observed in lateral occipito-temporal channels, while more pronounced responses to houses were seen in medial occipital channels. Yet, contrary to our expectation, we did not observe any modulatory effect of OT on these stimulus-driven neural responses, not in terms of enhanced social processing nor in terms of generally enhanced information salience. Bayesian analyses further validated these null findings. Given the short recording time and the robust individual responses, this method offers a fast, objective and reliable approach to quantifying selective neural sensitivity for social versus non-social information.

## Figures and Tables

**Table 1:** Results of LMM analyses on neural responses with treatment condition (OT, PL), stimulus type (face, house) and ROI (LOT, ROT, MO) as fixed effects and session order (OT session first, PL session first) as nuisance covariate.

Effect	*F* Value	*p* Value	Post-Hoc Test	
Treatment condition	*F*(1, 31) = 0.03	*p* = 0.856	/	
Stimulus type	*F*(1, 30) = 0.16	*p* = 0.696	/	
ROI	*F*(2, 40) = 48.03	***p* < 0.001**	MO (2.20 µV) > LOT (1.22 µV), *p* < 0.001	
			MO (2.20 µV) > ROT (1.61 µV), *p* < 0.001	
			ROT (1.61 µV) > LOT (1.22 µV), *p* = 0.012	
Session order	*F*(1, 29) = 0.46	*p* = 0.501	/	
Treatment condition and stimulus type	*F*(1, 551) = 0.08	*p* = 0.775	/	
Treatment condition and ROI	*F*(2, 552) = 0.28	*p* = 0.759	/	
Stimulus type and ROI	*F*(2, 557) = 184.26	***p* < 0.001**	face	ROT > LOT, *p* = 0.009
				MO > LOT, *p* = 0.006
			house	MO > LOT, *p* < 0.001
				MO > ROT, *p* < 0.001
			in LOT in ROT	face > house, *p* = 0.022
				face > house, *p* < 0.001
			in MO	house > face, *p* < 0.001
Treatment condition, stimulus type and ROI	*F*(2, 553) = 0.24	*p* = 0.788	/	

## Data Availability

The datasets generated during and/or analyzed during the current study are available from the corresponding author on reasonable request.

## References

[1] Kosfeld M., Heinrichs M.L., Zak P.J., Fischbacher U., Fehr E. (2005). Oxytocin increases trust in humans. Nature.

[2] Zak P.J., Stanton A.A., Ahmadi S. (2007). Oxytocin Increases Generosity in Humans. PLoS ONE.

[3] De Dreu C.K.W., Greer L.L., Handgraaf M.J.J., Shalvi S., Van Kleef G.A., Baas M., Velden F.S.T., Van Dijk E., Feith S.W.W. (2010). The Neuropeptide Oxytocin Regulates Parochial Altruism in Intergroup Conflict Among Humans. Science.

[4] Declerck C.H., Boone C., Kiyonari T. (2010). Oxytocin and cooperation under conditions of uncertainty: The modulating role of incentives and social information. Horm. Behav..

[5] Baumgartner T., Heinrichs M., Vonlanthen A., Fischbacher U., Fehr E. (2008). Oxytocin Shapes the Neural Circuitry of Trust and Trust Adaptation in Humans. Neuron.

[6] Marsh A.A., Yu H., Pine D.S., Blair R.J.R. (2010). Oxytocin improves specific recognition of positive facial expressions. Psychopharmacology.

[7] Lischke A., Berger C., Prehn K., Heinrichs M., Herpertz S.C., Domes G. (2012). Intranasal oxytocin enhances emotion recognition from dynamic facial expressions and leaves eye-gaze unaffected. Psychoneuroendocrinology.

[8] Domes G., Heinrichs M., Michel A., Berger C., Herpertz S.C. (2007). Oxytocin Improves “Mind-Reading” in Humans. Biol. Psychiatry.

[9] Domes G., Steiner A., Porges S.W., Heinrichs M. (2013). Oxytocin differentially modulates eye gaze to naturalistic social signals of happiness and anger. Psychoneuroendocrinology.

[10] Guastella A.J., Mitchell P.B., Dadds M.R. (2008). Oxytocin Increases Gaze to the Eye Region of Human Faces. Biol. Psychiatry.

[11] Guastella A.J., Einfeld S.L., Gray K.M., Rinehart N.J., Tonge B.J., Lambert T.J., Hickie I.B. (2010). Intranasal Oxytocin Improves Emotion Recognition for Youth with Autism Spectrum Disorders. Biol. Psychiatry.

[12] Dadds M.R., Macdonald E., Cauchi A., Williams K., Levy F., Brennan J. (2013). Nasal Oxytocin for Social Deficits in Childhood Autism: A Randomized Controlled Trial. J. Autism Dev. Disord..

[13] Huang Y., Huang X., Ebstein R.P., Yu R. (2021). Intranasal oxytocin in the treatment of autism spectrum disorders: A multilevel meta-analysis. Neurosci. Biobehav. Rev..

[14] Bakermans-Kranenburg M.J., Van Ijzendoorn M.H. (2013). Sniffing around oxytocin: Review and meta-analyses of trials in healthy and clinical groups with implications for pharmacotherapy. Transl. Psychiatry.

[15] Feifel D., MacDonald K., Cobb P., Minassian A. (2012). Adjunctive intranasal oxytocin improves verbal memory in people with schizophrenia. Schizophr. Res..

[16] Simeon D., Bartz J., Hamilton H., Crystal S., Braun A., Ketay S., Hollander E. (2011). Oxytocin administration attenuates stress reactivity in borderline personality disorder: A pilot study. Psychoneuroendocrinology.

[17] Shamay-Tsoory S.G., Fischer M., Dvash J., Harari H., Perach-Bloom N., Levkovitz Y. (2009). Intranasal Administration of Oxytocin Increases Envy and Schadenfreude (Gloating). Biol. Psychiatry.

[18] Taylor S.E., Gonzaga G.C., Klein L.C., Hu P., Greendale G.A., Seeman T.E. (2006). Relation of Oxytocin to Psychological Stress Responses and Hypothalamic-Pituitary-Adrenocortical Axis Activity in Older Women. Psychosom. Med..

[19] Tabak B.A., McCullough M.E., Szeto A., Mendez A.J., McCabe P.M. (2011). Oxytocin indexes relational distress following interpersonal harms in women. Psychoneuroendocrinology.

[20] Groppe S.E., Gossen A., Rademacher L., Hahn A., Westphal L., Gründer G., Spreckelmeyer K.N. (2013). Oxytocin Influences Processing of Socially Relevant Cues in the Ventral Tegmental Area of the Human Brain. Biol. Psychiatry.

[21] Shamay-Tsoory S.G., Abu-Akel A. (2016). The Social Salience Hypothesis of Oxytocin. Biol. Psychiatry.

[22] Rimmele U., Hediger K., Heinrichs M., Klaver P. (2009). Oxytocin Makes a Face in Memory Familiar. J. Neurosci..

[23] Unkelbach C., Guastella A.J., Forgas J.P. (2008). Oxytocin Selectively Facilitates Recognition of Positive Sex and Relationship Words. Psychol. Sci..

[24] Hu J., Qi S., Becker B., Luo L., Gao S., Gong Q., Hurlemann R., Kendrick K.M. (2015). Oxytocin selectively facilitates learning with social feedback and increases activity and functional connectivity in emotional memory and reward processing regions. Hum. Brain Mapp..

[25] Norman G.J., Cacioppo J.T., Morris J.S., Karelina K., Malarkey W.B., DeVries A.C., Berntson G.G. (2010). Selective influences of oxytocin on the evaluative processing of social stimuli. J. Psychopharmacol..

[26] Tollenaar M.S., Chatzimanoli M., van der Wee N.J., Putman P. (2013). Enhanced orienting of attention in response to emotional gaze cues after oxytocin administration in healthy young men. Psychoneuroendocrinology.

[27] Domes G., Sibold M., Schulze L., Lischke A., Herpertz S.C., Heinrichs M. (2012). Intranasal oxytocin increases covert attention to positive social cues. Psychol. Med..

[28] Baum A., Sachidanandam R., García-Sastre A. (2011). Different Amygdala Subregions Mediate Valence- Related and Attentional Effects of Oxytocin in Humans. Proc. Natl. Acad. Sci. USA.

[29] Leknes S., Wessberg J., Ellingsen D.-M., Chelnokova O., Olausson H., Laeng B. (2012). Oxytocin enhances pupil dilation and sensitivity to ‘hidden’ emotional expressions. Soc. Cogn. Affect. Neurosci..

[30] Hovey D., Martens L., Laeng B., Leknes S., Westberg L. (2020). The effect of intranasal oxytocin on visual processing and salience of human faces. Transl. Psychiatry.

[31] Andari E., Richard N., Leboyer M., Sirigu A. (2016). Adaptive coding of the value of social cues with oxytocin, an fMRI study in autism spectrum disorder. Cortex.

[32] Kirsch P., Esslinger C., Chen Q., Mier D., Lis S., Siddhanti S., Gruppe H., Mattay V.S., Gallhofer B., Meyer-Lindenberg A. (2005). Oxytocin Modulates Neural Circuitry for Social Cognition and Fear in Humans. J. Neurosci..

[33] Peltola M.J., Strathearn L., Puura K. (2018). Oxytocin promotes face-sensitive neural responses to infant and adult faces in mothers. Psychoneuroendocrinology.

[34] Huffmeijer R., Alink L.R., Tops M., Grewen K.M., Light K.C., Bakermans-Kranenburg M.J., van Ijzendoorn M.H. (2013). The impact of oxytocin administration and maternal love withdrawal on event-related potential (ERP) responses to emotional faces with performance feedback. Horm. Behav..

[35] Tillman R., Gordon I., Naples A., Rolison M., Leckman J.F., Feldman R., Pelphrey K.A., McPartland J.C. (2019). Oxytocin Enhances the Neural Efficiency of Social Perception. Front. Hum. Neurosci..

[36] Adrian E.D., Matthews B.H.C. (1934). The berger rhythm: Potential changes from the occipital lobes in man. Brain.

[37] Norcia A.M., Appelbaum L., Ales J.M., Cottereau B.R., Rossion B. (2015). The steady-state visual evoked potential in vision research: A review. J. Vis..

[38] Vettori S., Dzhelyova M., Van der Donck S., Jacques C., Van Wesemael T., Steyaert J., Rossion B., Boets B. (2019). Combined frequency-tagging EEG and eye tracking reveal reduced social bias in boys with autism spectrum disorder. Cortex.

[39] Vettori S., Dzhelyova M., Van Der Donck S., Jacques C., Steyaert J., Rossion B., Boets B. (2020). Frequency-Tagging Electroencephalography of Superimposed Social and Non-Social Visual Stimulation Streams Reveals Reduced Saliency of Faces in Autism Spectrum Disorder. Front. Psychiatry.

[40] Pei F., Pettet M.W., Norcia A.M. (2002). Neural correlates of object-based attention. J. Vis..

[41] Chen Y., Seth A.K., Gally J.A., Edelman G.M. (2003). The power of human brain magnetoencephalographic signals can be modulated up or down by changes in an attentive visual task. Proc. Natl. Acad. Sci. USA.

[42] Müller M.M., Andersen S., Trujillo N.J., Valdés-Sosa P., Malinowski P., Hillyard S.A. (2006). Feature-selective attention enhances color signals in early visual areas of the human brain. Proc. Natl. Acad. Sci. USA.

[43] Andersen S.K., Fuchs S., Müller M.M. (2011). Effects of Feature-selective and Spatial Attention at Different Stages of Visual Processing. J. Cogn. Neurosci..

[44] Störmer V.S., Winther G.N., Li S.-C., Andersen S.K. (2013). Sustained Multifocal Attentional Enhancement of Stimulus Processing in Early Visual Areas Predicts Tracking Performance. J. Neurosci..

[45] Baldauf D., Desimone R. (2014). Neural Mechanisms of Object-Based Attention. Science.

[46] de Heering A., Rossion B. (2015). Rapid categorization of natural face images in the infant right hemisphere. eLife.

[47] Van der Donck S., Dzhelyova M., Vettori S., Thielen H., Steyaert J., Rossion B., Boets B. (2019). Fast Periodic Visual Stimulation EEG Reveals Reduced Neural Sensitivity to Fearful Faces in Children with Autism. J. Autism Dev. Disord..

[48] Van Der Donck S., Dzhelyova M., Vettori S., Mahdi S.S., Claes P., Steyaert J., Boets B. (2020). Rapid neural categorization of angry and fearful faces is specifically impaired in boys with autism spectrum disorder. J. Child Psychol. Psychiatry.

[49] Vettori S., Dzhelyova M., Van der Donck S., Jacques C., Steyaert J., Rossion B., Boets B. (2018). Reduced neural sensitivity to rapid individual face discrimination in autism spectrum disorder. NeuroImage: Clin..

[50] Leleu A., Favre E., Yailian A., Fumat H., Klamm J., Amado I., Baudouin J.-Y., Franck N., Demily C. (2019). An implicit and reliable neural measure quantifying impaired visual coding of facial expression: Evidence from the 22q11.2 deletion syndrome. Transl. Psychiatry.

[51] Poncet F., Baudouin J.-Y., Dzhelyova M.P., Rossion B., Leleu A. (2019). Rapid and automatic discrimination between facial expressions in the human brain. Neuropsychologia.

[52] Van der Donck S., Moerkerke M., Dlhosova T., Vettori S., Dzhelyova M., Alaerts K., Boets B. (2022). Monitoring the effect of oxytocin on the neural sensitivity to emotional faces via frequency-tagging EEG: A double-blind, cross-over study. Psychophysiol..

[53] Grill-Spector K., Weiner K.S., Kay K., Gomez J. (2017). The Functional Neuroanatomy of Human Face Perception. Annu. Rev. Vis. Sci..

[54] Haxby J.V., Hoffman E.A., Gobbini M.I. (2000). The distributed human neural system for face perception. Trends Cogn. Sci..

[55] Kanwisher N., McDermott J., Chun M. (1997). The Fusiform Face Area: A Module in Human Extrastriate Cortex Specialized for Face Perception. J. Neurosci..

[56] Puce A., Allison T., Gore J.C., McCarthy G. (1995). Face-sensitive regions in human extrastriate cortex studied by functional MRI. J. Neurophysiol..

[57] Epstein R., Kanwisher N. (1998). A cortical representation of the local visual environment. Nature.

[58] Kadipasaoglu C.M., Conner C.R., Whaley M.L., Baboyan V.G., Tandon N. (2016). Category-Selectivity in Human Visual Cortex Follows Cortical Topology: A Grouped icEEG Study. PLoS ONE.

[59] Weiner K.S., Grill-Spector K. (2010). Sparsely-distributed organization of face and limb activations in human ventral temporal cortex. NeuroImage.

[60] Jacques C., Retter T.L., Rossion B. (2016). A single glance at natural face images generate larger and qualitatively different category-selective spatio-temporal signatures than other ecologically-relevant categories in the human brain. NeuroImage.

[61] Domes G., Lischke A., Berger C., Grossmann A., Hauenstein K., Heinrichs M., Herpertz S.C. (2010). Effects of intranasal oxytocin on emotional face processing in women. Psychoneuroendocrinology.

[62] Macdonald K.S. (2013). Sex, Receptors, and Attachment: A Review of Individual Factors Influencing Response to Oxytocin. Front. Behav. Neurosci..

[63] Graustella A.J., MacLeod C. (2012). A critical review of the influence of oxytocin nasal spray on social cognition in humans: Evidence and future directions. Horm. Behav..

[64] Quintana D.S., Lischke A., Grace S., Scheele D., Ma Y., Becker B. (2020). Advances in the field of intranasal oxytocin research: Lessons learned and future directions for clinical research. Mol. Psychiatry.

[65] Daughters K., Manstead A., Hubble K., Rees A., Thapar A., Van Goozen S.H.M. (2015). Salivary Oxytocin Concentrations in Males following Intranasal Administration of Oxytocin: A Double-Blind, Cross-Over Study. PLoS ONE.

[66] Striepens N., Kendrick K.M., Hanking V., Landgraf R., Wüllner U., Maier W., Hurlemann R. (2013). Elevated cerebrospinal fluid and blood concentrations of oxytocin following its intranasal administration in humans. Sci. Rep..

[67] Retter T.L., Rossion B. (2016). Uncovering the neural magnitude and spatio-temporal dynamics of natural image categorization in a fast visual stream. Neuropsychologia.

[68] Makeig S., Bell A.J., Jung T.-P., Sejnowski T.J., Touretzky D.S., Mozer M.C., Hasselmo M.E. (1996). Independent Component Analysis of Electroencephalographic Data. Advances in Neural Information Processing Systems 8.

[69] Regan D. (1989). Human Brain Electrophysiology: Evoked Potentials and Evoked Magnetic Fields in Science and Medicine.

[70] Rossion B., Torfs K., Jacques C., Liu-Shuang J. (2015). Fast periodic presentation of natural images reveals a robust face-selective electrophysiological response in the human brain. J. Vis..

[71] Dzhelyova M., Jacques C., Rossion B. (2016). At a Single Glance: Fast Periodic Visual Stimulation Uncovers the Spatio-Temporal Dynamics of Brief Facial Expression Changes in the Human Brain. Cereb. Cortex.

[72] Liu-Shuang J., Norcia A.M., Rossion B. (2013). An objective index of individual face discrimination in the right occipito-temporal cortex by means of fast periodic oddball stimulation. Neuropsychologia.

[73] Singmann H., Bolker B., Westfall J., Aust F., Ben- Shachar M.S. (2022). afex: Analysis of Factorial Experiments. R Package Version 1.1-0. https://CRAN.R-project.org/package=afex.

[74] R Core Team (2018). R: A Language and Environment for Statistical Computing.

[75] Lenth R. (2022). emmeans: Estimated Marginal Means, Aka Least-Squares Means. R Package Version 1.7.3. https://CRAN.R-project.org/package=emmeans.

[76] Morey R.D. (2021). and Rouder, J.N. BayesFactor: Computation of Bayes Factors for Common Designs. R Package Version 0.9.12-4.3. https://CRAN.R-project.org/package=BayesFactor.

[77] Lee M.D., Wagenmakers E.-J. (2014). Bayesian Model Comparison. Bayesian Cognitive Modeling: A Practical Course.

[78] Jeffreys H. (1961). Theory of Probabilit.

[79] Faul F., Erdfelder E., Lang A.-G., Buchner A. (2007). G*Power 3: A flexible statistical power analysis program for the social, behavioral, and biomedical sciences. Behav. Res. Methods.

[80] Hagen S., Jacques C., Maillard L., Colnat-Coulbois S., Rossion B., Jonas J. (2020). Spatially Dissociated Intracerebral Maps for Face- and House-Selective Activity in the Human Ventral Occipito-Temporal Cortex. Cereb. Cortex.

[81] Epstein R.A., Kveraga K., Bar M. (2014). Neural Systems for Visual Scene Recognition. Scene Vision: Making Sense of What We See.

[82] Jacques C., Witthoft N., Weiner K.S., Foster B.L., Rangarajan V., Hermes D., Miller K.J., Parvizi J., Grill-Spector K. (2015). Corresponding ECoG and fMRI category-selective signals in human ventral temporal cortex. Neuropsychologia.

[83] Dzhelyova M., Jacques C., Dormal G., Michel C., Schiltz C., Rossion B. (2019). High test-retest reliability of a neural index of rapid automatic discrimination of unfamiliar individual faces. Vis. Cogn..

[84] Jeste S.S., Frohlich J., Loo S.K. (2015). Electrophysiological biomarkers of diagnosis and outcome in neurodevelopmental disorders. Curr. Opin. Neurol..

[85] Winterton A., Westlye L.T., Steen N.E., Andreassen O.A., Quintana D.S. (2020). Improving the precision of intranasal oxytocin research. Nat. Hum. Behav..

[86] Tabak B.A., Teed A.R., Castle E., Dutcher J.M., Meyer M.L., Bryan R., Irwin M.R., Lieberman M.D., Eisenberger N.I. (2019). Null results of oxytocin and vasopressin administration across a range of social cognitive and behavioral paradigms: Evidence from a randomized controlled trial. Psychoneuroendocrinology.

[87] Bartz J.A., Zaki J., Bolger N., Hollander E., Ludwig N., Kolevzon A., Ochsner K.N. (2010). Oxytocin Selectively Improves Empathic Accuracy. Psychol. Sci..

[88] Feeser M., Fan Y., Weigand A., Hahn A., Gärtner M., Böker H., Grimm S., Bajbouj M. (2015). Oxytocin improves mentalizing–Pronounced effects for individuals with attenuated ability to empathize. Psychoneuroendocrinology.

[89] Harari-Dahan O., Bernstein A. (2014). A general approach-avoidance hypothesis of Oxytocin: Accounting for social and non-social effects of oxytocin. Neurosci. Biobehav. Rev..

[90] Harari-Dahan O., Bernstein A. (2017). Oxytocin attenuates social and non-social avoidance: Re-thinking the social specificity of Oxytocin. Psychoneuroendocrinology.

[91] Alaerts K., Taillieu A., Daniels N., Soriano J.R., Prinsen J. (2021). Oxytocin enhances neural approach towards social and non-social stimuli of high personal relevance. Sci. Rep..

[92] Trilla I., Drimalla H., Bajbouj M., Dziobek I. (2020). The Influence of Reward on Facial Mimicry: No Evidence for a Significant Effect of Oxytocin. Front. Behav. Neurosci..

[93] Melkonyan A., Liu L., Brown E.C., Meyer W., Madipakkam A.R., Ringelmann L., Lange F., Schmid S.M., Münte T.F., Park S.Q. (2020). Unchanged food approach-avoidance behaviour of healthy men after oxytocin administration. J. Neuroendocr..

[94] Lane A., Luminet O., Nave G., Mikolajczak M. (2016). Is there a Publication Bias in Behavioural Intranasal Oxytocin Research on Humans? Opening the File Drawer of One Laboratory. J. Neuroendocr..

[95] Bürkner P.-C., Williams D.R., Simmons T.C., Woolley J.D. (2017). Intranasal Oxytocin May Improve High-Level Social Cognition in Schizophrenia, But Not Social Cognition or Neurocognition in General: A Multilevel Bayesian Meta-analysis. Schizophr. Bull..

[96] Huang M., Liu K., Wei Z., Feng Z., Chen J., Yang J., Zhong Q., Wan G., Kong X.-J. (2021). Serum Oxytocin Level Correlates With Gut Microbiome Dysbiosis in Children With Autism Spectrum Disorder. Front. Neurosci..

[97] Peled-Avron L., Abu-Akel A., Shamay-Tsoory S. (2020). Exogenous effects of oxytocin in five psychiatric disorders: A systematic review, meta-analyses and a personalized approach through the lens of the social salience hypothesis. Neurosci. Biobehav. Rev..

[98] Ma Y., Shamay-Tsoory S., Han S., Zink C.F. (2016). Oxytocin and Social Adaptation: Insights from Neuroimaging Studies of Healthy and Clinical Populations. Trends Cogn. Sci..

[99] Walum H., Waldman I.D., Young L.J. (2015). Statistical and Methodological Considerations for the Interpretation of Intranasal Oxytocin Studies. Biol. Psychiatry.

